# How to estimate heritability: a guide for genetic epidemiologists

**DOI:** 10.1093/ije/dyac224

**Published:** 2022-11-25

**Authors:** Ciarrah-Jane S Barry, Venexia M Walker, Rosa Cheesman, George Davey Smith, Tim T Morris, Neil M Davies

**Affiliations:** Medical Research Council Integrative Epidemiology Unit, University of Bristol, Bristol, UK; Population Health Sciences, Bristol Medical School, University of Bristol, Bristol, UK; Medical Research Council Integrative Epidemiology Unit, University of Bristol, Bristol, UK; Population Health Sciences, Bristol Medical School, University of Bristol, Bristol, UK; Department of Surgery, University of Pennsylvania Perelman School of Medicine, Philadelphia, USA; PROMENTA Research Center, Department of Psychology, University of Oslo, Oslo, Norway; Medical Research Council Integrative Epidemiology Unit, University of Bristol, Bristol, UK; Population Health Sciences, Bristol Medical School, University of Bristol, Bristol, UK; Medical Research Council Integrative Epidemiology Unit, University of Bristol, Bristol, UK; Population Health Sciences, Bristol Medical School, University of Bristol, Bristol, UK; Medical Research Council Integrative Epidemiology Unit, University of Bristol, Bristol, UK; Population Health Sciences, Bristol Medical School, University of Bristol, Bristol, UK; K.G. Jebsen Center for Genetic Epidemiology, Department of Public Health and Nursing, NTNU, Norwegian University of Science and Technology, Trondheim, Norway

**Keywords:** Human genetics, epidemiologic methods, heritability, twin study, human genome, genome-wide association study

## Abstract

Traditionally, heritability has been estimated using family-based methods such as twin studies. Advancements in molecular genomics have facilitated the development of methods that use large samples of (unrelated or related) genotyped individuals. Here, we provide an overview of common methods applied in genetic epidemiology to estimate heritability, i.e. the proportion of phenotypic variation explained by genetic variation. We provide a guide to key genetic concepts required to understand heritability estimation methods from family-based designs (twin and family studies), genomic designs based on unrelated individuals [linkage disequilibrium score regression, genomic relatedness restricted maximum-likelihood (GREML) estimation] and family-based genomic designs (sibling regression, GREML-kinship, trio-genome-wide complex trait analysis, maternal-genome-wide complex trait analysis, relatedness disequilibrium regression). We describe how heritability is estimated for each method and the assumptions underlying its estimation, and discuss the implications when these assumptions are not met. We further discuss the benefits and limitations of estimating heritability within samples of unrelated individuals compared with samples of related individuals. Overall, this article is intended to help the reader determine the circumstances when each method would be appropriate and why.

Key Messages

- A range of methods exists to estimate heritability—the genetic contributions to epidemiological phenotypes, each with strengths and limitations.
- Understanding these is important to correctly interpret results from genetic epidemiology studies and weigh (or counterweigh) evidence appropriately.
- Methods for estimating heritability should be carefully considered concerning the underlying assumptions, biases and suitability of the data.
- Common issues across methods developed to estimate heritability include bias due to population stratification, epistasis and assortative mating.
- Genomic and non-genomic methods using samples of related individuals can account for unobserved environmental confounding.

## Introduction

Many human phenotypes are influenced by a complex mix of genetic and environmental factors. Therefore, it is important to comprehensively account for genetic influence when discerning how phenotypic variation in the population arises. The broad role of genetics is commonly quantified using heritability—the proportion of phenotypic variation that genetic variation can statistically explain (see Box 1).

Box 1: Defining heritability Heritability: The proportion of phenotypic variation for a specific measurement that can be attributed to genetic variation. Narrow-sense heritability is solely an estimate of additive genetic effects—the summed effects of multiple genes contributing to a single phenotype.^1^ Broad-sense heritability is an estimate of both additive and non-additive genetic effects, and thus encompasses the additive, dominance and epistatic genetic effects^1^^,^^2^ (see Supplementary Box S1, available as Supplementary data at *IJE* online). Estimates of heritability cannot necessarily be translated into a wider or more general population as it is specific to the population within the sample.

There is evidence that most phenotypes are heritable, with heritability typically being higher for biological proximal, e.g. eye colour, than social or behavioural traits, e.g. extraversion.^3^^,^^4^

Methods to estimate heritability from samples of related individuals capitalize on the known shared genetic variance between relatives, e.g., offspring share half of the genome of each of their parents. Path analysis has been widely implemented, assuming a linear variance model. This separates the phenotype of interest into components: the additive genetic (A), common familial environment (C) and the environmental contribution unique to the individual (E), termed the ACE model.^5^ However, these methods require a range of assumptions about the cause of similarity within family pairs. The increasing availability of large samples of genotyped individuals stimulated the development of methods to estimate heritability within samples of unrelated individuals.^6^ These methods capitalize on the large sample size and minimal environmental bias between unrelated individuals. However, these approaches are limited to capturing the additive component of the ACE model. Further, we note that bias could occur if genetic similarity was correlated with environmental similarity and the environment was not accurately measured. Thus, a portion of heritability is likely not captured by estimates. These techniques have recently been extended to incorporate samples of related individuals, simultaneously accounting for the common environment and using the wealth of available genotyped data in studies of unrelated individuals.^7^

It has been theorized that a further portion of heritability, as estimated by some genomic methods, is not captured as the contributing single-nucleotide polymorphisms (SNPs) are rare^8^ (see Supplementary Box S1, available as Supplementary data at *IJE* online). These SNPs are not detected in genome-wide association studies as they require much larger sample sizes for their effects to be reliably estimated.^8^^,^^9^

Many methods exist to estimate heritability, which require different testable and untestable assumptions. These estimators can be influenced by demographic, familial and genomic factors such as population stratification, indirect genetic effects, assortative mating, linkage disequilibrium (LD) and epistasis (see Supplementary Box S1, available as Supplementary data at *IJE* online).^6^ Estimates of heritability are specific to the population under study and may not be transferable across different populations across space or time. Heritability estimates generally fall into two categories: broad-sense and narrow-sense. ‘Broad-sense heritability’ is the proportion of phenotypic variation statistically explained by total genetic variation, including dominance and epistasis (see Supplementary Box S1, available as Supplementary data at *IJE* online). ‘Narrow-sense heritability’ refers to phenotypic variation explained by additive genetic variation only.^2^ Heritability may be used to estimate both continuous and binary traits. Conventionally, to enable this estimation for binary traits, an underlying continuous liability is assumed.^10^ This additional assumption alters the precise definition of the binary heritability measurement to be the proportion of variance on the latent liability scale due to genetic variation.^11^^,^^12^

**Figure 1:**
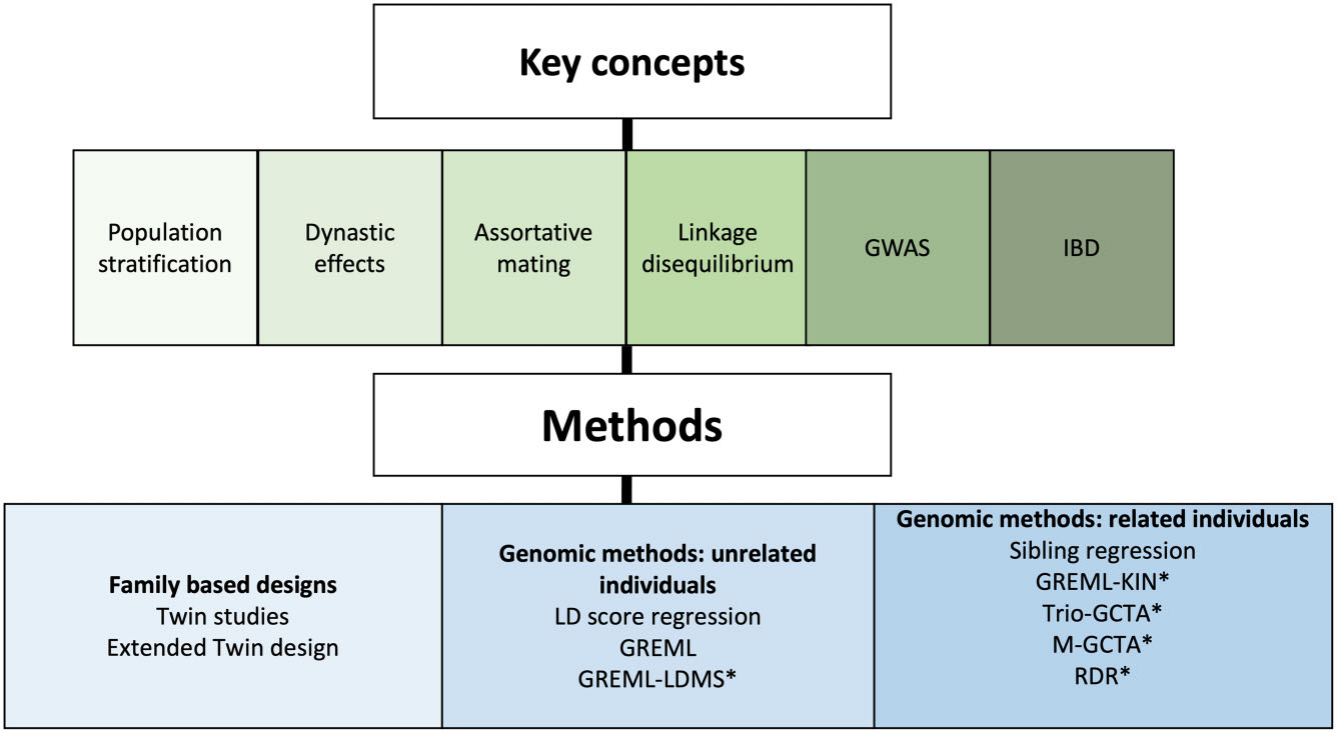
A flowchart detailing the outline of the review. * indicates methods that can be found in the Supplementary Notes (available as Supplementary data at *IJE* online)

Here, we describe methods for estimating heritability, their assumptions and their biases. We have selected approaches that are widely implemented in the literature and group them into three sections (Figure 1): (i) family-based designs, (ii) genomic designs based on unrelated individuals and (iii) family-based molecular genomic designs. We review these methods and approaches to estimate heritability and discuss their respective benefits and limitations. Definitions and key concepts that may aid conceptual understanding are available in Supplementary Box S1 (available as Supplementary data at *IJE* online).

## Family-based designs

Family-based designs use samples of closely related individuals, e.g. siblings or parents and offspring. Family-based designs can estimate heritability even if molecular genetic data are unavailable.

### Twin studies

Twin studies examine phenotypic differences between monozygotic (MZ) twins, who are genetically identical at conception, and dizygotic (DZ) twins, who, on average, share 50% of their segregating genetic variation,^13^ the genetic variation that results in individual differences between the pair.^13^ If MZ twins are more phenotypically similar than DZ twins, then this suggests that genetic variation affects phenotypes.^14^ The classic twin design is also known as the ACE model. The ACE model estimates narrow-sense heritability, assuming the dominance genetic variance (see Supplementary Box S1, available as Supplementary data at *IJE* online) is zero. However, by implementing an ADE model [in which variance components are modelled as additive (A), dominance and epistasis (D) and non-shared environmental effects], estimated heritability may be partitioned into additive and dominance variance components. Notably, to the extent that MZ and DZ twins experience parental indirect genetic effects similarly, these effects will be included within the common environment (C) component. Twin studies may also be used to study gene–environment interactions.^16^ Twin studies require several assumptions:

- The shared environment makes an equal contribution to the phenotype of interest across both MZ and DZ twin pairs, termed the ‘equal environment assumption’ (EEA).^2^^,^^7^^,^^15^^,^^17^ If the EEA is invalid, heritability estimates are likely to be inflated because different environments would be mistakenly attributed to differences in genetic variation;^15^ see Supplementary Note 1 (available as Supplementary data at *IJE* online) for more information about the validity of the EEA.
- ‘Twins are generalizable to the general population’ regarding the phenotype of interest. The validity of this assumption has been demonstrated in multiple studies.
- ‘Random mating’ occurs within the population.^13^^,^^18^
- The ‘confounding role of environmental similarity’ on genetic factors and outcomes is limited (see Supplementary Box S1, available as Supplementary data at *IJE* online).^19^

If all these assumptions hold, then comparing the phenotypic correlations of MZ and DZ twins can reliably estimate heritability.^13^ See Supplementary Note 2 (available as Supplementary data at *IJE* online) for the extended twin design.

## Genomic methods: unrelated individuals

The mass characterization of the human genome has generated a rich, vast resource for genetics research.^20^ Measured genomic methods refer to statistical methods applied to molecular genetic (including whole-genome) data, which have either been directly measured or imputed using reference panels. Some measured genome methods focus on estimating ‘SNP heritability’—a particular case of narrow-sense heritability estimated from measured SNPs.

### LD score regression

Linkage disequilibrium score regression (here LDSR, but sometimes referred to as LDSC) is a regression-based method that can separate genetic and confounding effects and estimate SNP heritability.^21^ The LD score is a measure of how well each SNP can tag other local SNPs. SNPs with high LD scores are more likely to tag causal SNPs and thus have a larger association on average than SNPs with low LD scores.^21^ An LD score is created within a population reference panel for each SNP to represent the amount of tagged genetic variation explained by the SNP (see Supplementary Box S1, available as Supplementary data at *IJE* online). The reference panel accounts for some LD structure within a given population, which means population stratification bias can be separated from genuine polygenicity. Summary statistics from multiple SNPs are regressed on the LD score of each SNP of interest, with the estimated intercept quantifying the bias. The LDSR slope estimates the variance of the phenotype explained by all SNPs (i.e. the heritability) used to estimate the LD scores.^21^ Thus, LDSR enables inflated genome-wide association study (GWAS) (see Supplementary Box S1, available as Supplementary data at *IJE* online) test statistics to be differentiated from confounding bias. Software has been created for the implementation of LD score regression (see Supplementary Box S2, available as Supplementary data at *IJE* online). SNPs may tag both individual large and multiple weak effects, in contrast to other genome-wide methods. However, LDSR estimates are less accurate where fewer SNPs are available. LDSR depends on several assumptions:

- The ‘variance explained per SNP is uncorrelated with the LD score’. Therefore, rare SNPs are assumed to have larger effect sizes than common SNPs.^22^ However, this may not hold in all circumstances, such as phenotypes with correlated LD scores and minor allele frequency (MAF) (Supplementary Box S1, available as Supplementary data at *IJE* online).^21^
- The ‘target sample of interest is well matched to the LD reference panel’.^21^ If this does not hold, the accuracy of estimates decreases as the genetic heterogeneity discrepancy between the reference and sample of interest increases. However, population stratification resulting from genetic drift does not correlate with LD and cannot be distinguished by LDSR.

### Genomic relatedness restricted maximum likelihood

**Figure 2:**
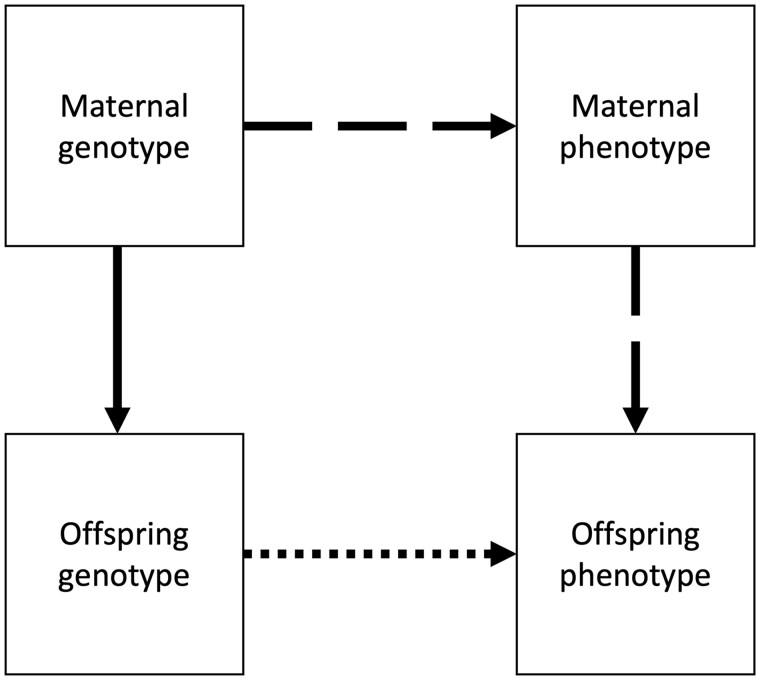
An illustration demonstrating genetic effects between a mother and offspring. We demonstrate the indirect maternal genetic effects between mother and offspring (maternal phenotype to offspring phenotype, dashed arrow), direct maternal genetic effects (bold arrow) and the direct genetic offspring effect on itself (dotted arrow). Note these effects also hold for the paternal genotype, alongside effects resulting from the likely parental genotype correlation

Genomic relatedness restricted maximum likelihood (GREML) estimates SNP heritability from measured genomic data. Notably, the method uses unrelated individuals who are assumed to vary randomly in their genetic similarity. The technique capitalizes on the logic that more genetically related individuals are more phenotypically similar in a population of unrelated people. Practically, this method is implemented by constructing a genetic relationship matrix (GRM) capturing the degree of relatedness between every pair of individuals at every SNP location (see Supplementary Box S1, available as Supplementary data at *IJE* online).^17^ The extent that the genetic matrix predicts phenotypic similarity reflects the degree of heritability. Note, heritability estimates employing a GRM depend on large sample sizes. There is a range of software available to implement GREML (see Supplementary Box S2, available as Supplementary data at *IJE* online). The following assumptions are required for GREML estimation:

- ‘All genetic effects are direct’ (i.e. biological effects in the offspring), even though transmitted variants can also influence phenotypes indirectly via the parents. If indirect genetic effects exist, these will be attributed to direct effects, as illustrated in Figure 2.Consequently, GREML overestimates the contribution of direct genetic effects (see Supplementary Box S1, available as Supplementary data at *IJE* online) and inflates heritability estimates of specific phenotypes in the presence of indirect effects.^7^
- ‘Individuals do not share environmental influences’. The restriction to unrelated individuals (typical threshold <0.025) limits confounding by common environmental effects alongside reducing contamination of non-additive genetic effects.^6^^,^^17^^,^^23^ Additionally, GREML assumes no epistatic effects and all estimated genetic effects are assumed to be additive.
- ‘GREML only captures direct, additive effects of common SNPs’ and not rare genetic effects or non-additive genetic effects. SNP-based methods may therefore be used to validate estimates derived from family-based methods requiring alternative assumptions. Thus, as with other methods using measured SNP data, estimates obtained via GREML can be considered to provide a lower bound of heritability.^8^ However, this lower bound should be used with caution as there are potential sources of inflation within GREML estimates that would not occur in family-based design studies, e.g. within GREML any common environmental effects that are not explicitly modelled are likely to be reflected in heritability estimates.
- There is ‘random mating’, meaning that GREML heritability estimates may be biased in the presence of assortative mating because of directional LD between SNPs (see Supplementary Box S1 and Supplementary Note 3, available as Supplementary data at *IJE* online).^17^
- There are ‘strong assumptions about genetic architecture’, i.e. the characteristics of genetic variation that cause heritable phenotypic variability.^24^ GREML assumes that SNP genotypes are standardized, with normally distributed effects independent of LD. Thus, SNPs with lower MAF are implicitly assumed to have a larger per-allele effect as variants with larger effect sizes typically have lower MAF.^6^
- The ‘true genetic effect variance–covariance structure between pairs of individuals is known’. This is an estimate derived from the GRM rather than the true genetic effect variance–covariance structure.^6^ This estimate is reasonable in samples of unrelated individuals as genetic and environmental similarities are assumed to be independent. However, this may be violated in the twin study setting.

In the presence of population stratification, standard GREML methods are likely to overestimate heritability. Specific GREML population-based methods include an adjustment for the first principal components of the estimated kinship matrix into the model with fixed effects to account for population stratification.^17^^,^^23^^,^^25^ However, these are not thought to control for the bias induced by population stratification comprehensively, so some bias remains.^2^ Additionally, GREML methods are sensitive to LD. GREML heritability estimates reflect LD between SNPs and unmeasured genetic variation. As a result, estimates using data from sparse SNP arrays with relatively few SNPs will be smaller than estimates from data with a denser array.^6^ Furthermore, common SNPs may not tag less-common SNPs well.

The maximum LD correlation will decrease as the difference in MAF increases. Heritability estimates will be biased when SNPs are located in genomic regions with different LD properties to the rest of the genome [e.g. if regions of high LD, such as the human leukocyte antigen (HLA), the complex of genes coding the proteins that regulate the human immune system, are particularly important for a phenotype].^26^

In addition, GREML is highly sensitive to uneven LD, over- and under-estimating heritability in high and low LD areas, respectively. This is because the correlation between SNPs distorts their estimated contribution to heritability; see Supplementary Notes 4 and 5 (available as Supplementary data at *IJE* online) for methods to overcome this limitation.

An extension to GREML using unrelated individuals has been developed. In LD- and MAF-stratified GREML (GREML-LDMS), effects of less-common variants can be estimated through the use of LD and MAF data on imputed SNPs. For details on this method, see Supplementary Notes 4 and 5 (available as Supplementary data at *IJE* online).

## Genomic methods: related individuals

A shared limitation of the genomic methods applied to unrelated individuals is an inability to account for environmental confounding (see Supplementary Box S1, available as Supplementary data at *IJE* online). Various methods have been developed to enable the implementation of genomic methods in large samples of related individuals.

### Sibling regression

Identity-by-descent (IBD) (see Supplementary Box S1, available as Supplementary data at *IJE* online) can be used to estimate narrow-sense heritability in samples of individuals who are usually, but not always closely related.^14^^,^^27^ Linear mixed modelling using IBD kinship matrices can be used to estimate the additive genetic effect.

Sibling regression is an approach to estimate heritability derived from IBD. On average, siblings are 50% genetically similar. Variation around this 50% average exists due to random segregations at conception, which are approximately independent of most environmental effects (including indirect genetic effects).^7^ Hence, within sibling pairs, if genetic variation affects a phenotype, siblings who are more similar and share more of their genomes IBD should be more phenotypically similar. Note estimates obtained from siblings capture less of the genetic variance than estimates obtained by twin studies. Heritability estimates obtained via sibling regression do not include indirect genetic effects as this method is limited to narrow-sense heritability.

Statistical methods using sibling regression to estimate heritability are dependent on the following assumptions:

- ‘Estimates must also account for regions of LD’.^28^
- ‘Estimates of the percentage of shared IBD between siblings across the entire genome are proportional to the number of causal additive SNPs between siblings’ for the trait of interest.^27^
- ‘Siblings who have inherited an IBD segment from a common ancestor have identical genetic segments in that region’. Hence estimates include the rare variant effects in that region (except de novo mutation and other variant-introducing events).^29^
- ‘The additive genetic covariance between relatives is proportional to the proportion of the genome that is shared IBD’.^27^
- There is ‘random mating’. However, sibling regression estimates may be inflated by assortative mating as the induced phenotypic correlation increases the genetic and phenotypic variation in the population, alongside relative pairwise phenotypic covariances. Thus, the common environmental effect between individuals could be overestimated and result in inflated heritability estimates unless assortative mating is accounted for.^15^

It is possible to implement GREML to estimate the heritability of binary traits, such as in case–control studies.^23^ This requires additional assumptions:

- The trait has an ‘underlying normally distributed threshold liability model’. Note that this is a statistically untestable assumption. This has caused some controversy in interpreting heritability estimates for binary traits within the literature.^30^^,^^31^
- The ‘estimate of the variance’ that may be ‘apportioned to the SNPs’ on the ‘binary scale’ may be ‘linearly transformed to a continuous liability scale’.

The authors note here that adjustment for ascertainment bias is necessary and any element of the data that may cause allele frequencies between the cases and controls to differ greater than that expected under the null hypothesis may produce spurious estimates as within-group relatedness (e.g. case–case members) will be greater than between groups (e.g. case–control members). Therefore, when implementing GREML with binary traits, stringent quality control of data is required.^23^ Further, some argue that setting an artificial risk threshold to determine the disease status may create mathematically meaningless results.^30^^,^^31^

The following four methods—GREML-kinship (GREML-KIN), maternal-genome-wide complex trait analysis (M-GCTA), Trio-GCTA and relatedness disequilibrium regression (RDR)—are extensions to GREML using related individuals. GREML-KIN estimates heritability whilst controlling for the shared environment by including relatives. M-GCTA, Trio-GCTA and RDR are similar approaches as the phenotypic variance of the offspring is decomposed into direct and indirect genetic effects by including one or both parental genotypes. See Supplementary Notes 6–9 (available as Supplementary data at *IJE* online) for details on GREML-KIN, M-GCTA, Trio-GCTA and RDR.

We provide a summary of the advantages and disadvantages of the methods discussed in Table 1.

## Conclusion

A range of methods is available to estimate heritability using family-based and genomic designs. Each approach has distinct advantages and limitations, such as controlling bias due to population stratification in related individuals or the wide availability of large-sample GWAS data. Triangulation of heritability estimates across different methods with different assumptions within samples is likely to provide the most robust evidence for the heritability of phenotypes, with consideration for any expected estimand differences.

**Table 1:** A summary of the strengths and limitations of each discussed method

Method	Strengths	Limitations
Twin studies	Estimates not substantially affected by violation of the EEA assumption; provide an upper-bound estimate of heritability; incorporating the effects of rare SNPs; within-pair effects not impacted by population stratification	Estimates may be biased upwards due to shared environmental effects interacting with additive genetic effects; cannot determine the effect of epistatic interactions; estimates of the common environmental effects inflated by assortative mating
Extended twin studies^a^	May differentiate the additive and non-additive components of genetic variance, in addition to the effects of assortative mating	Cannot account for inflation in estimates of non-additive effects
Sibling regression	Can provide an estimate of narrow-sense heritability; no additional assumptions about the distribution of SNP effects; incorporates the effects of rare SNPs into heritability estimates; robust to genotyping errors and some missingness; partially accounts for population stratification	Large sample size required for precise results due to the small standard deviation of IBD shared between siblings, e.g. Visscher *et al.* determined the average proportion of the genome-shared IBD within sibling pairs to have a standard deviation of 0.036 within their sample; the estimate is relative to a chosen reference population^1^
LD score regression	Computationally efficient as it only requires summary-level data; partially accounts for population structure, see Appendix; precision increases with a greater number of SNPs; if the variance explained by each SNP is uncorrelated with LD score, estimates are unbiased (contingent on other assumptions); possible to implement using free online tool^21^	Rare SNPs are assumed to have a larger effect; accuracy of estimates is dependent on a well-matched population panel; cannot estimate total heritability; a large sample is required to have reasonable power when detecting SNPs with lower heritability
GREML	Provides a ‘lower-bound’ estimate for heritability; possible to implement using widely available software; partially accounts for population stratification; can be implemented in large-scale biobanks of unrelated individuals	Cannot distinguish direct and indirect genetic effects, which may inflate estimates; samples restricted to genotyped individuals; estimates inflated by assortative mating; additional assumptions about SNP effect sizes required; not suited to estimate the contribution of rare SNPs; estimates are highly sensitive to LD
GREML-LDMS^a^	Reduced bias resulting from LD compared with GREML-SC	Less precise estimates relative to other GREML methods
GREML-KIN^a^	Estimate heritability along with shared environmental effects coming from siblings, parents and spouses	Requires samples with ranging relatedness; effect estimates may still be confounded by unmodelled shared environmental factors; greater power needed to detect smaller effects
Trio-GCTA^a^	Able to account for indirect genetic effects within heritability estimates; assumptions about the structure of LD are not necessary; partially accounts for population stratification	Requires genotyped parent–offspring trios; additional assumptions about the distribution of genetic and residual effects are necessary; assortative mating and epistatic effects will bias estimates
M-GCTA^a^	Able to account for indirect genetic effects within heritability estimates; possible to implement using freely available software	Requires genotyped maternal or paternal–offspring pairs; may be biased by assortative mating; large sample size required; cannot simultaneously account for both parental genotypes
RDR^a^	Environmental effects are not included in heritability estimates; arguably less constrictive assumptions are required than for alternative genome-wide family methods, such as the distribution of SNP effects; simulations have demonstrated population stratification can be mostly accounted for;^11^ a greater proportion of variance from rare SNPs may be captured relative to other genome-wide methods	Epistasis and assortative mating will distort estimates; samples cannot contain individuals related by direct descent; population stratification may modify the assumed IBD-sharing relationship

EEA, equal environment assumption; GCTA, genome-wide complex trait analysis; GREML, genomic relatedness restricted maximum-likelihood estimation; GREML-KIN, genomic relatedness restricted maximum-likelihood estimation kinship; IBD, identity-by-descent; LD, linkage disequilibrium; M-GCTA, maternal-genome-wide complex trait analysis; RDR, relatedness disequilibrium regression; SNP, single-nucleotide polymorphism.

a Indicates methods that can be found in the Supplementary Notes (available as Supplementary data at *IJE* online).

## Ethics approval

Ethics approval not needed for this study containing no participants.

## Supplementary Material

dyac224_Supplementary_Data

## Data Availability

N/A.
