# Correction to: How to estimate heritability: a guide for genetic epidemiologists

**DOI:** 10.1093/ije/dyae039

**Published:** 2024-03-19

This is a correction to: Ciarrah-Jane S Barry, Venexia M Walker, Rosa Cheesman, George Davey Smith, Tim T Morris, Neil M Davies, How to estimate heritability: a guide for genetic epidemiologists, *International Journal of Epidemiology*, Volume 52, Issue 2, April 2023, Pages 624–632, https://doi.org/10.1093/ije/dyac224

In the originally published version of this manuscript, there are two errors:

The following sentence,

“Methods to estimate heritability from samples of related individuals capitalize on the (average) shared genetic variance between relatives, e.g. offspring share broadly half of their genotype with siblings.”

Has been updated to read:

“Methods to estimate heritability from samples of related individuals capitalize on the known shared genetic variance between relatives, e.g., offspring share half of the genome of each of their parents.”

The following sentence,

“Identity-by-descent (IBD) (see Supplementary Box S1, available as Supplementary data at *IJE* online) can estimate narrow-sense heritability in samples of individuals who are not closely related.”

Has been updated to read:

“Identity-by-descent (IBD) (see Supplementary Box S1, available as Supplementary data at *IJE* online) can be used to estimate narrow-sense heritability in samples of individuals who are usually, but not always, closely related.”

These errors have been corrected.

