# Supplementary material for: How to estimate heritability: a guide for genetic epidemiologists
Source: Int J Epidemiol. 2022 Nov 25;52(2):624–32. doi: 10.1093/ije/dyac224 (PMC10114051; doi:10.1093/ije/dyac224)
Supplement: dyac224_Supplementary_Data [file dyac224_supplementary_data.docx]

# SUPPLEMENTARY BOXES

## Box S1: Key concepts and definitions

| **Key concepts** | | |
| --- | --- | --- |
| Common, low-frequency and rare SNPs | We define SNPs with minor allele frequency (MAF) > 5% to be common SNPs, SNPs with MAF 1-5% to be low-frequency SNPs, and SNPs with MAF < 1% to be rare SNPs ^1, 2^. | |
| Epistasis | Epistasis is the effect of interaction between genes at multiple locations ^3, 4^. Epistasis alters the variance of a phenotype ^5, 6^. The effects between one variant and a variant in a different stratum of a single parameter can have effects in opposing directions. Hence the genetic effects of the parameter may not be detectable if one gene is counteracting the expression of the other ^7^. There is not yet a consensus on the importance of epistasis. Some argue epistatic effects play a minor role in phenotypic expression relative to additive effects ^8-11^, yet others propose epistasis may greatly impact upon estimates ^12-15^ which would consequently influence estimates of heritability obtained via methods that do not account for it ^6, 16, 17^. Current studies appear to only detect interactions with very large effects; therefore, it is possible many more interactions exist but are undetectable ^18-22^. | |
| Dominance | Dominance refers to allelic interactions within a gene ^23^. Thus, dominance genetic variation results in phenotypic differences that result from allelic interactions. Dominance variation has been found to contribute little to SNP based estimates of heritability ^24^. | |
| Population stratification | Population stratification refers to differences in allele frequencies across groups within a population ^25-27^. These allele frequency differences can arise from non-random mating and geographic separation. Members of isolated populations experience random changes in allele frequency over time (genetic drift), which can lead to observable differences in allele frequency over many generations ^28^. It is widely recognized that population stratification can inflate estimates of heritability, although this is method dependent. For example, in some within-family genetic methods the estimate for heritability is not affected method ^27, 29-32^. | |
| Direct genetic effects | Direct genetic effects are the effects of an individual’s own genotype on their own phenotype ^33^. | |
| Indirect parental genetic effects | Indirect parental genetic effects occur when parental genotype affects offspring phenotype through its expression in the parental phenotype ^34, 35^. For example, parents with more education-associated alleles may procure more books for their households than parents with fewer education associated alleles (the phenotypic expression of the parents’ genetic variation). This may in turn positively influence their offspring’s reading ability or knowledge, resulting in higher offspring educational outcomes. Indirect parental genetic effects can inflate heritability estimated within the offspring generation in certain situations. Indirect genetic effects are a similar concept to genetic nurture, dynastic effects, and, within twin studies literature, passive gene-environment correlation ^36, 37^. | |
| Assortative mating | Assortative mating occurs when people choose mates non-randomly with respect to their phenotypes. Assortative mating can be single phenotype (e.g., education to education) or cross phenotype (e.g., education to height). Assortative mating can induce correlations between phenotypes and genetic variation for different phenotypes ^34, 38, 39^. Thus, when assortative mating is present, heritable traits become non-randomly distributed as partners will be more, or less, genetically similar than expected by chance which may invalidate key assumptions required by the method. | |
| Linkage disequilibrium | Linkage disequilibrium (LD) is the non-random association of alleles at different loci within a population. Loci are in LD when the frequency of association of their different alleles is higher or lower than would be expected by chance ^40^. Genome-wide LD conveys a population’s history, for example geographic subdivision. Genomic region LD reflects the gene-frequency evolution, including mutation, natural selection, and gene conversion ^40, 41^. LD can either be local, i.e. correlations between genetic variants that are in similar positions in the genome and are inherited in a block, or non-local in which more widely spaced genetic variants are correlated ^42^. | |
| Identity-by-descent and identity-by-state | Identify-by-descent (IBD) is a fundamental concept defining genetic relatedness between individuals ^43^. Two or more individuals who have the same genotype at a given point in the genome are identity-by-state (IBS). Further, individuals are IBD if they are IBS and have inherited this variation from a common ancestor. Therefore, the difference between IBS and IBD stems from knowledge of the relationship between the sequence and its origin ^44^, illustrated in Figure B1. The covariance between the percentage of the genome shared between related pairs in a population (e.g., siblings or cousins) and their phenotypic similarity can be estimated using IBD segments discovered by dense genotyping, which covers a wide range of SNPs in a population ^16, 45, 46^. Heritability estimation methods that use IBD as a measure of relatedness have been developed ^47^.  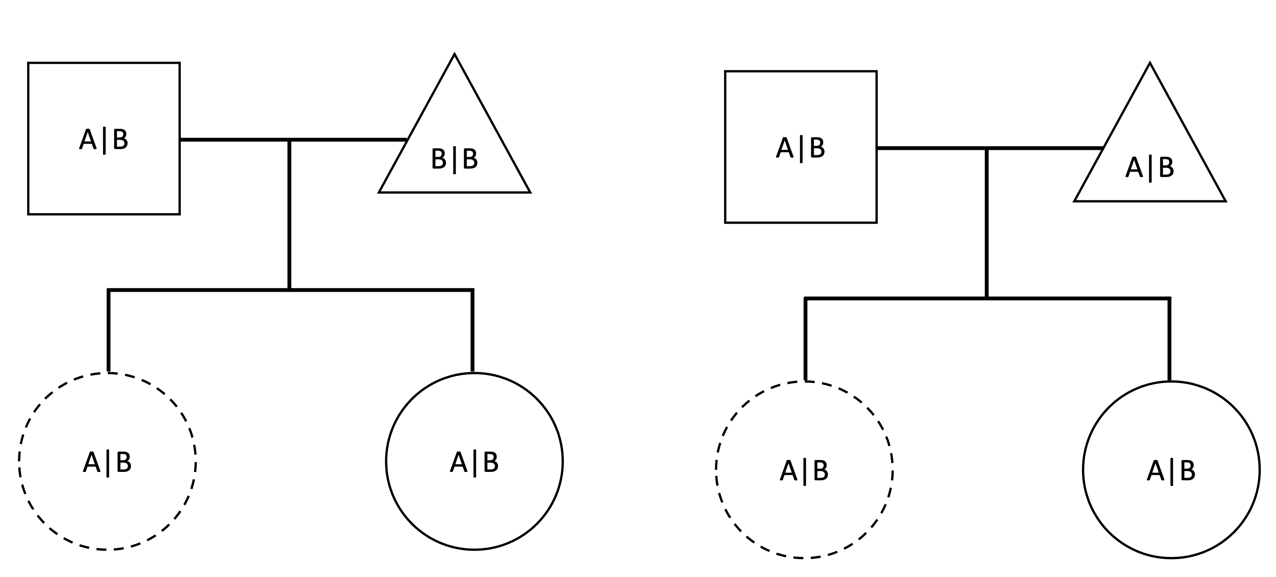  Figure B1: Identity-by-descent (IBD), left: On the left, the two offspring (dashed and solid circles) have inherited allele A from their mother (square). This means they are both IBS (i.e., they have the same genotype) and are also IBD (i.e., they both inherited A from their mother). Identity-by-state (IBS), right: On the right, both offspring have inherited an A allele. However, the dashed circle inherited A allele from their mother (square) and the solid circle inherited the A allele from their father (triangle). Thus, the siblings on the left are IBS only (i.e., they have the same genotype but have inherited the alleles from different parents). | |
| Genome-wide association studies | Genome-wide association studies (GWAS) estimate millions of SNP-phenotype associations in large samples, vastly accelerating the study of the genetic architecture of complex phenotypes ^48^. Individual SNPs generally explain a small proportion of phenotypic variability as the magnitude of association is small. Hence, associations for many SNPs in a GWAS may not reach the genome-wide significance threshold $\boldsymbol{p\times}\boldsymbol{10}^{\boldsymbol{-8}}$ in available samples ^49-51^. This strict p-value threshold is required because of multiple testing and to reduce false positive discoveries ^52^. Most phenotypes and diseases are influenced by many genetic and environmental factors (known as the omnigenic model of inheritance) ^53, 54^. Many genomic methods to estimate heritability are reliant upon data obtained from GWAS ^55^. | |
| Confounding | A confounder is a variable that fulfils the following criteria: 1) it is associated with the exposure of interest, 2) it is associated with the outcome when conditioning on the exposure, 3) it is not associated with the outcome except through the exposure of interest.  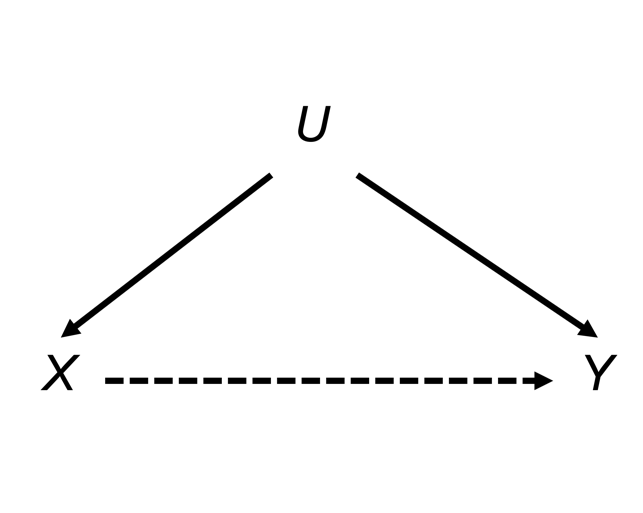  Figure B2: A directed acyclic graph demonstrating the concept of confounding. The relationship of interest between the exposure (X) and outcome (Y) is demonstrated by the dashed line. The directional arrows from the variable (U) to both the exposure and outcome demonstrate confounding, as U is a common cause of both the exposure and outcome. Therefore, estimates of the association between the exposure and outcome are biased due to the unaccounted presence of the confounding variable. | |
| Environmental confounding | Environmental confounding refers to confounding variables in the relationship between an exposure and outcome that are found in the environment, or environmental factors ^56^. | |
| **Definitions** | | |
| **Term** | | **Definition** |
| Population structure | | When regional or geographic differences exist in the distribution of SNPs or the allele frequency related to the trait of interest ^57^. |
| GWAS summary statistics | | Sharing large amounts of sensitive individual level genotypic and phenotypic data is often impractical. However, GWAS summary statistics such as SNP specific effect size estimates, standard errors and allele frequencies are readily available, with many found in online databases. Summary level data is non-identifiable and has much lower computational costs, reducing the risk of to security and improving efficiency. In addition, the use of open-access data within a study aids replication which is an essential part of SNP based analyses ^58^. Note, restricting analysis to summary level only does limit potential downstream analysis as less information is available. |
| Population reference panel | | A set of individuals of a specific population that have been selected and defined be the “standard” genotype of that population. Typically, the ancestry of these individuals originates from a singular location to ensure good genetic representation of that area, and a single representative would be selected if closely related individuals are initially included. |
| Genetic relatedness matrix | | For a sample of n individuals, the GRM is of dimension $n\times n$ whereby each element of the matrix is the pairwise-relatedness coefficient between two individuals. |
| Ascertainment bias | | When the sample obtained is not a random sample of the genetic landscape of the population meaning certain members of the population have a higher or lower sampling probability relative to others, thus the sample is biased ^59^. |
| Directional linkage disequilibrium | | Linkage disequilibrium (LD) is the non-random association of alleles between nearby genetic variants at a specific point in a population. If these alleles occur with greater frequency than should be expected by chance, then they are in LD. LD has been orientated with respect to genetic frequencies such that $LD=p_{\mathrm{AB}}p_{\mathrm{ab}}-p_{\mathrm{Ab}}p_{\mathrm{aB}}<0$ where A,a,B,b are the alleles at the linked loci with respective frequencies $p_{A}{,p}_{a}, p_{B},p_{b}$ and assign A and B the alleles with the higher frequency ^60^. |
| Minor allele frequency | | The frequency of the minor allele for a given SNP in a specific population. |

Box S2: Related software to implement specific methods

| **Heritability estimation method** | **Software** | **Description** |
| --- | --- | --- |
| LD Score regression | LDSC ^61^ | Estimates LD Scores and variance components from summary statistics. |
| GREML | GCTA Software tool ^55^ | Estimates the proportion of phenotypic variance explained by all genome-wide SNPs on phenotypic traits. |
| GREML-LDMS | GCTA-LDMS ^62^ | Estimates heritability using whole genome sequence data whilst correcting for LD bias. |
| M-GCTA | M-GCTA Software package ^63^ | Estimates the proportion of variance explained by maternal/paternal/offspring genotypes on offspring phenotypes. |
| Trio-GCTA | Trio-GCTA ^64^ | Estimates the proportion of variance explained whilst separating the direct and indirect familial genetic effects. |

# SUPPLEMENTARY NOTES

## Twin studies: the validity of the EEA

The validity of the EEA has been tested within the literature through a comparison of trait similarity as a function of independently assessed zygosity and family-perceived zygosity, with respect to psychiatric disorders. Consistent with previous findings, little evidence was found to suggest perceived zygosity influences twin resemblance. Hence, differing expectations contingent on zygosity were not determined to substantially bias twin study findings and violation of the EEA will have little effect ^65^. Further, simulations have demonstrated the impact of a violation of the EEA is likely to be modest, ranging between a 7-14% reduction in heritability estimate ^66^.

## Extended twin designs

Extended twin designs model the similarity between other relatives alongside the twins. This enables additional parameters to be estimated. For example, it is possible to determine the twin-specific and non-parental components of the shared environment alongside the additive and non-additive components of genetic variance ^67^. Additionally, the effect of assortative mating may be accounted for by including spouses ^68^.

## GREML: the assumption of random mating

Non-random selection increases, or decreases, additive genetic variation through the gametic phase disequilibrium. This means gene alleles are no longer randomly associated as SNPs occur with a frequency greater, or less, than the product of the frequency of the two relevant alleles, as would be expected through random mating ^69^. For example, GREML likely overestimates the contribution of genetic variation for phenotypes that are impacted by assortative mating, e.g., intelligence ^70^.

## GREML: methods to overcome correlation between SNPs

LD adjusted kinship (LDAK) has been proposed to reduce bias and increase precision in these circumstances. This calculates a SNP weight in relation to how well it is tagged by genomic neighbours ^71^. Weighting estimates were found to largely eliminate bias relative to estimates calculated using standard kinship matrix, however effects may be overstated. Alternative methods address LD heterogeneity but are computationally intensive ^62, 72^.

1. **GREML-LDMS**

SNP based estimates of heritability can be biased when LD differs between causal variants and other variants. However, it has been demonstrated that this bias may be reduced by stratifying GREML heritability estimates based on a joint model of LD and MAF, termed GREML-LDMS ^73^. This is a multi-component approach in which SNPs are imputed by their MAF and regional LD. LD scores are calculated for each segment, and from this SNPs are stratified into quartiles. A GRM is then calculated from the stratified SNPs for each sample and a REML analysis is carried out using multiple GRMs ^74^. Simulations have demonstrated GREML-LDMS heritability estimates to be less biased compared to other GREML based estimation methods, though they are less precise ^72, 73^. Software is available to implement GREML-LDMS, see Appendix Box S2.

GREML-LDMS estimates require the following set of assumptions regarding the GRM to hold. First, that there is no gene-environment correlation. Second, that SNP effects are normally distributed, independent of LD and inversely proportional to MAF. Third, a much larger sample size (>30,000) is required for GREML-LDMS than the standard GREML approach ^72^.

## GREML-KIN

GREML-KIN is a modification of GREML that enables the estimation of additional genetic and shared environmental effects amongst family members ^75^. Specifically, estimates of heritability can be segregated into the variance corresponding to family-specific genetic effects, and genetic effects at the population level. Two GRMs are included, one capturing the genetic relatedness of family members, and the other capturing genetic similarity among unrelated individuals. Matrices indexing environmental sharing are included to control for the fact that familial genetic similarity is correlated with environmental similarity ^75^. Since LD is stronger between more related individuals (compared with less related individuals), the family relatedness matrix in GREML-KIN may capture effects of variation not tagged by common SNPs. However, relative to other methods using the same sample size, smaller effects are harder to detect in GREML-KIN. For example, if the GRM contributes less that 5% of the overall phenotypic variance, only the major contributing components to the trait will be estimated reliably ^75, 76^. Further, GREML-KIN requires a sample comprised of a sufficient number of participants with ranging relatedness. Whether a dataset contains sufficient participants for GREML-KIN can be determined via simulations ^76^.

## M-GCTA

Maternal-GCTA (M-GCTA) estimates the proportion of offspring phenotypic variation that can be explained by both maternal and offspring genetic variation ^63^. The covariance between the offspring genetic effect on offspring phenotype, and the maternal genetic effect on maternal phenotype, is used to estimate the contribution of the indirect maternal effect to the offspring phenotype of interest ^63^. Therefore, within M-GCTA the maternal effects include the indirect effects of the maternally provided environment (including intrauterine effects), which would typically bias standard GREML estimates ^63, 77^. M-GCTA requires genetic information from large samples of mother-offspring (or father-offspring) pairs to model indirect genetic effects ^77, 78^, which may limit sample size compared to GREML in unrelated individuals. A further limitation to the method is the inability to simultaneously account for both parental genotypes, thus estimates may be biased by the genetic effects of the parent omitted in the model ^63^. There is a software package available to implement M-GCTA, see Appendix Box S2.

Most assumptions of standard GREML are common to M-GCTA, however M-GCTA does not rest upon assumptions of sample relatedness. M-GCTA additionally assumes that variance due to indirect maternal effects and correlations between direct and indirect maternal effects are assumed to be non-zero ^55, 63^.

## Trio-GCTA

Trio-GCTA is very similar to M-GCTA however it includes genomic information from both parents ^64^. The presence of indirect effects from both parents can be tested empirically with samples of mother-father-offspring trios. The calculation of the direct and indirect genetic effects can apply to any member of the trio, although interpretation of parameters is specific to the individual of interest ^60^. It has been stated it is unclear how assortative mating could influence estimates, however we note the direct effect estimates from Trio-GCTA are robust to both population stratification and assortative mating ^38, 64, 79^. The main limitation of trio-GCTA is the requirement for large samples of genotyped parent offspring trios. Most assumptions of standard GREML are common to Trio-GCTA, excluding the assumption of sample unrelatedness and assumptions about the structure of LD within the sample. Violation of the random mating assumption has an undetermined impact on inferences from Trio-GCTA at present ^64, 70^. In addition, Trio-GCTA has strict requirements about the distribution of the genetic and residual effects, and assumes they are independent, identically distributed and follow a multivariate normal distribution.

## Relatedness disequilibrium regression

RDR estimates the influence of the indirect genetic parental effect on the offspring on top of the direct genetic effect, akin to separating out unmeasurable heritable phenotypes ^27, 80^. RDR differentiates the direct genetic effects from the indirect based on the random segregation during meiosis. Specifically, it allows us to decompose the elements of the phenotypic variation into direct genetic and other effects (e.g., environmental) and exploit the independence of offspring genotype and environment conditional on parental genotype ^81^. This is likely to hold within pairs of individuals who are not related by direct descent as they are unlikely to affect one another’s residual environments. Here consistency is not affected by environmental confounding. Note, population stratification may bias estimates of parental indirect genetic effects. An alternative was to apply RDR is through the use of IBS segments, however the estimates produced are very similar ^27^.

Specific assumptions must be met within this method. First, direct genetic effects are assumed to be additive, e.g., no epistasis occurs. It may be possible to incorporate non-additive genetic effects within estimates, but consideration must be given to the corresponding non-additive associations between parental genotype and environment to ensure residual environmental effects are uncorrelated with the non-additive effects ^27^. Second, we have random mating, with analogous reasoning to previous methods. Third, for consistency of the estimator it is necessary that the trios are independent. This prevents segregation events within the individual pairs’ parents becoming dependent on each other, which could induce bias comparable to the proportion of direct descendent related pairs ^27^.

Whilst GREML-KIN can distinguish heritability and shared familial environmental effects, it is not a within family-specific method and therefore heritability estimates may still be confounded by indirect genetic effects.

**References**

1. Sun X, Namkung J, Zhu X, Elston RC. Capability of common SNPs to tag rare variants. *BMC proceedings* 2011; **5 Suppl 9**: S88-S.

2. Bomba L, Walter K, Soranzo N. The impact of rare and low-frequency genetic variants in common disease. *Genome Biol* 2017; **18**: 77.

3. Hemani G, Shakhbazov K, Westra H-J, et al. Detection and replication of epistasis influencing transcription in humans. *Nature* 2014; **508**: 249-53.

4. de Visser JAGM, Cooper TF, Elena SF. The causes of epistasis. *Proc Biol Sci* 2011; **278**: 3617-24.

5. Cordell HJ. Epistasis: what it means, what it doesn't mean, and statistical methods to detect it in humans. *Human Molecular Genetics* 2002; **11**: 2463-8.

6. Rose AM, Bell LCK. Epistasis and immunity: the role of genetic interactions in autoimmune diseases. *Immunology* 2012; **137**: 131-8.

7. Browman KE, Crabbe JC. Alcoholism: Genetic Aspects. In: Smelser NJ, Baltes PB, editors. *International Encyclopedia of the Social & Behavioral Sciences*. Oxford: Pergamon; 2001. p. 371-8.

8. Crow JF. On epistasis: why it is unimportant in polygenic directional selection. *Philos Trans R Soc Lond B Biol Sci* 2010; **365**: 1241-4.

9. Hill WG, Goddard ME, Visscher PM. Data and theory point to mainly additive genetic variance for complex traits. *PLoS Genet* 2008; **4**: e1000008-e.

10. Hill WG. "Conversion" of epistatic into additive genetic variance in finite populations and possible impact on long-term selection response. *J Anim Breed Genet* 2017; **134**: 196-201.

11. Mäki-Tanila A, Hill WG. Influence of gene interaction on complex trait variation with multilocus models. *Genetics* 2014; **198**: 355-67.

12. Shao H, Burrage LC, Sinasac DS, et al. Genetic architecture of complex traits: large phenotypic effects and pervasive epistasis. *Proc Natl Acad Sci U S A* 2008; **105**: 19910-4.

13. Carter AJR, Hermisson J, Hansen TF. The role of epistatic gene interactions in the response to selection and the evolution of evolvability. *Theoretical Population Biology* 2005; **68**: 179-96.

14. Malmberg RL, Mauricio R. QTL-based evidence for the role of epistasis in evolution. *Genetical Research* 2005; **86**: 89-95.

15. Nelson RM, Pettersson ME, Carlborg Ö. A century after Fisher: time for a new paradigm in quantitative genetics. *Trends in Genetics* 2013; **29**: 669-76.

16. Zuk O, Hechter E, Sunyaev SR, Lander ES. The mystery of missing heritability: Genetic interactions create phantom heritability. *Proceedings of the National Academy of Sciences* 2012; **109**: 1193.

17. Polderman TJC, Benyamin B, de Leeuw CA, et al. Meta-analysis of the heritability of human traits based on fifty years of twin studies. *Nat Genet* 2015; **47**: 702-9.

18. Maller J, George S, Purcell S, et al. Common variation in three genes, including a noncoding variant in CFH, strongly influences risk of age-related macular degeneration. *Nat Genet* 2006; **38**: 1055-9.

19. Rioux JD, Xavier RJ, Taylor KD, et al. Genome-wide association study identifies new susceptibility loci for Crohn disease and implicates autophagy in disease pathogenesis. *Nat Genet* 2007; **39**: 596-604.

20. Barrett JC, Clayton DG, Concannon P, et al. Genome-wide association study and meta-analysis find that over 40 loci affect risk of type 1 diabetes. *Nat Genet* 2009; **41**: 703-7.

21. Strange A, Capon F, Spencer CC, et al. A genome-wide association study identifies new psoriasis susceptibility loci and an interaction between HLA-C and ERAP1. *Nat Genet* 2010; **42**: 985-90.

22. Evans DM, Spencer CC, Pointon JJ, et al. Interaction between ERAP1 and HLA-B27 in ankylosing spondylitis implicates peptide handling in the mechanism for HLA-B27 in disease susceptibility. *Nat Genet* 2011; **43**: 761-7.

23. Fisher RA. XV.—The correlation between relatives on the supposition of Mendelian inheritance. *Earth and Environmental Science Transactions of the Royal Society of Edinburgh* 1919; **52**: 399-433.

24. Zhu Z, Bakshi A, Vinkhuyzen AAE, et al. Dominance genetic variation contributes little to the missing heritability for human complex traits. *Am J Hum Genet* 2015; **96**: 377-85.

25. Singh A. Population Stratification. In: Gellman MD, Turner JR, editors. *Encyclopedia of Behavioral Medicine*. New York, NY: Springer New York; 2013. p. 1506-7.

26. Wacholder S, Rothman N, Caporaso N. Population Stratification in Epidemiologic Studies of Common Genetic Variants and Cancer: Quantification of Bias. *JNCI: Journal of the National Cancer Institute* 2000; **92**: 1151-8.

27. Young AI, Frigge ML, Gudbjartsson DF, et al. Relatedness disequilibrium regression estimates heritability without environmental bias. *Nat Genet* 2018; **50**: 1304-10.

28. Hellwege JN, Keaton JM, Giri A, Gao X, Velez Edwards DR, Edwards TL. Population Stratification in Genetic Association Studies. *Curr Protoc Hum Genet* 2017; **95**: 1.22.1-1..3.

29. Browning SR, Browning BL. Population structure can inflate SNP-based heritability estimates. *Am J Hum Genet* 2011; **89**: 191-5.

30. Dandine-Roulland C, Bellenguez C, Debette S, Amouyel P, Génin E, Perdry H. Accuracy of heritability estimations in presence of hidden population stratification. *Sci Rep* 2016; **6**: 26471-.

31. Price AL, Patterson NJ, Plenge RM, Weinblatt ME, Shadick NA, Reich D. Principal components analysis corrects for stratification in genome-wide association studies. *Nat Genet* 2006; **38**: 904-9.

32. Marchini J, Cardon LR, Phillips MS, Donnelly P. The effects of human population structure on large genetic association studies. *Nat Genet* 2004; **36**: 512-7.

33. Warrington NM, Hwang L-D, Nivard MG, Evans DM. Estimating direct and indirect genetic effects on offspring phenotypes using genome-wide summary results data. *Nature Communications* 2021; **12**: 5420.

34. Brumpton B, Sanderson E, Heilbron K, et al. Avoiding dynastic, assortative mating, and population stratification biases in Mendelian randomization through within-family analyses. *Nature Communications* 2020; **11**: 3519.

35. Baud A, McPeek S, Chen N, Hughes KA. Indirect Genetic Effects: A Cross-disciplinary Perspective on Empirical Studies. *Journal of Heredity* 2021.

36. Kong A, Thorleifsson G, Frigge ML, et al. The nature of nurture: Effects of parental genotypes. *Science* 2018; **359**: 424-8.

37. Plomin R, DeFries JC, Loehlin JC. Genotype-environment interaction and correlation in the analysis of human behavior. *Psychological Bulletin* 1977; **84**: 309-22.

38. Morris TT, Davies NM, Hemani G, Smith GD. Population phenomena inflate genetic associations of complex social traits. *Sci Adv* 2020; **6**: eaay0328-eaay.

39. Yengo L, Robinson MR, Keller MC, et al. Imprint of assortative mating on the human genome. *Nat Hum Behav* 2018; **2**: 948-54.

40. Slatkin M. Linkage disequilibrium — understanding the evolutionary past and mapping the medical future. *Nature Reviews Genetics* 2008; **9**: 477-85.

41. Devlin B, Risch N. A comparison of linkage disequilibrium measures for fine-scale mapping. *Genomics* 1995; **29**: 311-22.

42. Young AI, Benonisdottir S, Przeworski M, Kong A. Deconstructing the sources of genotype-phenotype associations in humans. *Science (New York, NY)* 2019; **365**: 1396-400.

43. Wright S. On the Probable Error of Mendelian Class Frequencies. *The American Naturalist* 1917; **51**: 373-5.

44. Stevens EL, Heckenberg G, Roberson EDO, Baugher JD, Downey TJ, Pevsner J. Inference of relationships in population data using identity-by-descent and identity-by-state. *PLoS Genet* 2011; **7**: e1002287-e.

45. Thompson EA. Identity by descent: variation in meiosis, across genomes, and in populations. *Genetics* 2013; **194**: 301-26.

46. Browning SR, Browning BL. High-resolution detection of identity by descent in unrelated individuals. *Am J Hum Genet* 2010; **86**: 526-39.

47. Browning SR, Browning BL. Identity-by-descent-based heritability analysis in the Northern Finland Birth Cohort. *Hum Genet* 2013; **132**: 129-38.

48. McCarthy MI, Abecasis GR, Cardon LR, et al. Genome-wide association studies for complex traits: consensus, uncertainty and challenges. *Nature Reviews Genetics* 2008; **9**: 356-69.

49. Manolio TA, Collins FS, Cox NJ, et al. Finding the missing heritability of complex diseases. *Nature* 2009; **461**: 747-53.

50. Nolte IM, Jansweijer JA, Riese H, et al. A Comparison of Heritability Estimates by Classical Twin Modeling and Based on Genome-Wide Genetic Relatedness for Cardiac Conduction Traits. *Twin Research and Human Genetics* 2017; **20**: 489-98.

51. Plomin R, DeFries JC, Knopik VS, Neiderhiser JM. Top 10 Replicated Findings From Behavioral Genetics. *Perspect Psychol Sci* 2016; **11**: 3-23.

52. Risch N, Merikangas K. The future of genetic studies of complex human diseases. *Science* 1996; **273**: 1516-7.

53. McCarthy MI, Hirschhorn JN. Genome-wide association studies: potential next steps on a genetic journey. *Human Molecular Genetics* 2008; **17**: R156-R65.

54. Boyle EA, Li YI, Pritchard JK. An Expanded View of Complex Traits: From Polygenic to Omnigenic. *Cell* 2017; **169**: 1177-86.

55. Yang J, Lee SH, Goddard ME, Visscher PM. GCTA: a tool for genome-wide complex trait analysis. *Am J Hum Genet* 2011; **88**: 76-82.

56. Vanderweele TJ, Ko Y-A, Mukherjee B. Environmental confounding in gene-environment interaction studies. *Am J Epidemiol* 2013; **178**: 144-52.

57. Haworth S, Mitchell R, Corbin L, et al. Apparent latent structure within the UK Biobank sample has implications for epidemiological analysis. *Nature Communications* 2019; **10**: 333.

58. Craig DW, Goor RM, Wang Z, et al. Assessing and managing risk when sharing aggregate genetic variant data. *Nature reviews Genetics* 2011; **12**: 730-6.

59. Heslot N, Rutkoski J, Poland J, Jannink J-L, Sorrells ME. Impact of marker ascertainment bias on genomic selection accuracy and estimates of genetic diversity. *PloS one* 2013; **8**: e74612-e.

60. Langley CH, Crow JF. The direction of linkage disequilibrium. *Genetics* 1974; **78**: 937-41.

61. Bulik-Sullivan BK, Loh P-R, Finucane HK, et al. LD Score regression distinguishes confounding from polygenicity in genome-wide association studies. *Nat Genet* 2015; **47**: 291-5.

62. Yang J, Bakshi A, Zhu Z, et al. Genetic variance estimation with imputed variants finds negligible missing heritability for human height and body mass index. *Nat Genet* 2015; **47**: 1114-20.

63. Qiao Z, Zheng J, Helgeland Ø, et al. Introducing M-GCTA a Software Package to Estimate Maternal (or Paternal) Genetic Effects on Offspring Phenotypes. *Behavior Genetics* 2020; **50**: 51-66.

64. Eilertsen EM, Jami ES, McAdams TA, et al. Direct and Indirect Effects of Maternal, Paternal, and Offspring Genotypes: Trio-GCTA. *Behavior Genetics* 2021; **51**: 154-61.

65. Kendler KS. Twin Studies of Psychiatric Illness: Current Status and Future Directions. *Archives of General Psychiatry* 1993; **50**: 905-15.

66. Felson J. What can we learn from twin studies? A comprehensive evaluation of the equal environments assumption. *Social Science Research* 2014; **43**: 184-99.

67. Eaves L, Heath A, Martin N, et al. Comparing the biological and cultural inheritance of personality and social attitudes in the Virginia 30 000 study of twins and their relatives. *Twin Research* 1999; **2**: 62-80.

68. Keller MC, Medland SE, Duncan LE, et al. Modeling extended twin family data I: description of the Cascade model. *Twin Res Hum Genet* 2009; **12**: 8-18.

69. Machado FB, de Vasconcellos Machado L, Bydlowski CR, Bydlowski SP, Medina-Acosta E. Gametic phase disequilibrium between the syntenic multiallelic HTG4 and HMS3 markers widely used for parentage testing in Thoroughbred horses. *Molecular Biology Reports* 2012; **39**: 1447-52.

70. Plomin R, Deary IJ. Genetics and intelligence differences: five special findings. *Mol Psychiatry* 2015; **20**: 98-108.

71. Speed D, Hemani G, Johnson Michael R, Balding David J. Improved Heritability Estimation from Genome-wide SNPs. *The American Journal of Human Genetics* 2012; **91**: 1011-21.

72. Evans LM, Tahmasbi R, Vrieze SI, et al. Comparison of methods that use whole genome data to estimate the heritability and genetic architecture of complex traits. *Nat Genet* 2018; **50**: 737-45.

73. Yang J, Zeng J, Goddard ME, Wray NR, Visscher PM. Concepts, estimation and interpretation of SNP-based heritability. *Nat Genet* 2017; **49**: 1304-10.

74. Diez-Fairen M, Bandres-Ciga S, Houle G, et al. Genome-wide estimates of heritability and genetic correlations in essential tremor. *Parkinsonism Relat Disord* 2019; **64**: 262-7.

75. Hill WD, Arslan RC, Xia C, et al. Genomic analysis of family data reveals additional genetic effects on intelligence and personality. *Mol Psychiatry* 2018; **23**: 2347-62.

76. Xia C, Amador C, Huffman J, et al. Pedigree- and SNP-Associated Genetics and Recent Environment are the Major Contributors to Anthropometric and Cardiometabolic Trait Variation. *PLoS Genet* 2016; **12**: e1005804-e.

77. Eaves LJ, Pourcain BS, Smith GD, York TP, Evans DM. Resolving the effects of maternal and offspring genotype on dyadic outcomes in genome wide complex trait analysis ("M-GCTA"). *Behavior genetics* 2014; **44**: 445-55.

78. Jami ES, Eilertsen EM, Hammerschlag AR, et al. Maternal and paternal effects on offspring internalizing problems: Results from genetic and family-based analyses. *Am J Med Genet B Neuropsychiatr Genet* 2020; **183**: 258-67.

79. Davies NM, Howe LJ, Brumpton B, Havdahl A, Evans DM, Davey Smith G. Within family Mendelian randomization studies. *Hum Mol Genet* 2019; **28**: R170-r9.

80. Visscher PM, Medland SE, Ferreira MAR, et al. Assumption-Free Estimation of Heritability from Genome-Wide Identity-by-Descent Sharing between Full Siblings. *PLoS Genet* 2006; **2**: e41.

81. Cheesman R, Eilertsen EM, Ahmadzadeh YI, et al. How important are parents in the development of child anxiety and depression? A genomic analysis of parent-offspring trios in the Norwegian Mother Father and Child Cohort Study (MoBa). *BMC Med* 2020; **18**: 284-.
